# Role of Cortisol and Testosterone in Risky Decision-Making: Deciphering Male Decision-Making in the Iowa Gambling Task

**DOI:** 10.3389/fnins.2021.631195

**Published:** 2021-06-15

**Authors:** Varsha Singh

**Affiliations:** Humanities and Social Sciences, Indian Institute of Technology, New Delhi, India

**Keywords:** Iowa gambling task, risk, male decision making, stress-cortisol, testosterone, dual hormone hypothesis

## Abstract

Despite the widely observed high risk-taking behaviors in males, studies using the Iowa gambling task (IGT) have suggested that males choose safe long-term rewards over risky short-term rewards. The role of sex and stress hormones in male decision-making is examined in the initial uncertainty and the latter risk phase of the IGT. The task was tested at peak hormone activity, with breath counting to facilitate cortisol regulation and its cognitive benefits. Results from IGT decision-making before and after counting with saliva samples from two all-male groups (breath *vs*. number counting) indicated that cortisol declined independent of counting. IGT decision-making showed phase-specific malleability: alteration in the uncertainty phase and stability in the risk phase. Working memory showed alteration, whereas inhibition task performance remained stable, potentially aligning with the phase-specific demands of working memory and inhibition. The results of hierarchical regression for the uncertainty and risk trials indicated that testosterone improved the model fit, cortisol was detrimental for decision-making in uncertainty, and decision-making in the risk trials was benefitted by testosterone. Cortisol regulation accentuated hormones’ phase-specific effects on decision-making. Aligned with the dual-hormone hypothesis, sex, and stress hormones might jointly regulate male long-term decision-making in the IGT.

## Introduction

In general, males display higher risk-taking behaviors than do females (Byrnes et al., 1999), while females display more risk aversion than do males (Charness and Gneezy, 2012). However, in a widely used decision-making task, the Iowa gambling task (IGT; Bechara et al., 1994), males outperform females by choosing safe long-term rewards over risky short-term rewards (Bolla et al., 2004; see review in van den Bos et al., 2013). Unknown to the decision maker, the task involves 100 trials of picking one card after another (forming five blocks of 20 trials) by deciding between four decks of cards labeled A, B, C, and D. Each card pick results in a reward and, at times, is accompanied by occasional losses. Decision-making is carried out based on intertemporality; that is, more cards are drawn from the two safe/good decks, C and D, that give a small immediate reward (50 points), but result in long-term net gain, whereas fewer cards are picked from risky/bad decks, A and B, that give large immediate rewards (100 points), but result in long-term net loss. In other words, deck cards (C and D) are safe/good in the long term because, although they give small immediate rewards of 50 points for each of the card picked, 50% of the cards drawn from deck C have a loss in the range of 25–75 points, and 10% of cards drawn from deck D have a loss of 250 points; therefore, drawing 10 cards from decks C and D results in a long-term net gain of 250 points. On the other hand, every card drawn from the risky/bad decks (A and B) is risky because they give a large immediate reward (100 points), but 50% of the cards drawn from deck A give a loss in the range of 75–100 points, and 10% of the cards drawn from deck B give a loss of 1,250 points; therefore, drawing 10 cards from the risky decks results in a long-term net loss of 250 points. Furthermore, the outcomes associated with the decks are largely unknown in the initial IGT phase (trials 1–40, blocks 1 and 2), and this phase is characterized by uncertainty, whereas, as the payoffs associated with each of the four decks become known with task progression (trials 60–100, blocks 4 and 5), the later trials are characterized as decision-making under risk (Brand et al., 2007). Long-term decision-making is prominent in the risk phase of male IGT decision-making (Stanton et al., 2011; van den Bos et al., 2013; Evans and Hampson, 2014). Male preference for long-term decision-making is observed in countries that vary in socioeconomic and gender inequality (Singh et al., 2020), pointing toward a potential biological basis of male IGT decision-making and risk-taking.

Hormones might play a critical role in male decision-making in the IGT; for instance, a prenatal male sex hormone, testosterone influences male risk-taking in the IGT (Reavis and Overman, 2001; d’Acremont and Van der Linden, 2006; van den Bos et al., 2013; Evans and Hampson, 2014), impairing decision-making in the IGT (Reavis and Overman, 2001; Stanton et al., 2011; Evans and Hampson, 2014). Although testosterone is higher in males compared to females (Southren et al., 1965), males make more long-term decisions in the IGT (van den Bos et al., 2013), and long-term decision-making is prominent when male testosterone is low (Stanton et al., 2011). Testosterone’s effects on male long-term decision-making might reflect a regulatory control, and one likely factor might be the stress hormone cortisol, which might inhibit testosterone’s effects on male IGT decision-making. Male risk-taking is governed by testosterone’s and cortisol’s combined effects (Mehta and Josephs, 2010). Since cortisol stress triggers motor impulsivity and a “fight-or-flight” response in males (Taylor et al., 2000), its regulation might play a role in testosterone’s effects on risk-taking in the IGT. For instance, cortisol impairs long-term decision-making in males (Preston et al., 2007) by increasing risk-taking in IGT decision-making (Simonovic et al., 2017). Cortisol regulation might aid in testosterone regulation for keeping male risk-taking under control; that is, the dual regulation of cortisol and testosterone might contribute to male IGT decision-making.

Furthermore, the effects of cortisol and testosterone might differ in the two phases of male IGT decision-making (uncertainty and risk phases). For instance, since cortisol impairs working memory and the impairment is more detrimental to male IGT decision makers (Preston et al., 2007; van den Bos et al., 2009), cortisol’s effect might be more prominent in the uncertainty phase of the IGT. Furthermore, cortisol elevation impaired male IGT decision-making, the most prominent impairment being in the uncertainty phase (Figure 2A in van den Bos et al., 2009), and the deficit was possibly due to cortisol impairment of higher working memory demands (Bagneux et al., 2013). Cortisol’s effect on male IGT decision-making in the risk phase is less clear: one study indicated that cortisol stress impaired male decision-making in the risk phase (Table 3 in Preston et al., 2007), while in another study, cortisol stress improved decision-making in the risk phase (fourth block; van den Bos et al., 2009). Unlike cortisol’s effect in the risk phase, testosterone’s most prominent effect is more clearly observed in the IGT risk phase (Evans and Hampson, 2014). Decision-making in the uncertainty phase was least affected by high testosterone; in contrast, decision-making in the risk phase was impaired by high testosterone and was benefited by low testosterone (Figure 2 in Stanton et al., 2011), suggesting that testosterone might influence male decision-making in the risk phase of the task.

To examine the phase-specific effects of testosterone and cortisol, IGT task performance was assessed during the morning period when testosterone levels are high (Kriegsfeld et al., 2002), and post-awakening cortisol activity as a reliable biomarker (Pruessner et al., 1997) reflects the peak cortisol elevation and decline (Kalsbeek et al., 2012). Morning cortisol elevation reflects post-awakening activation response, whereas post-awakening cortisol decline reflects cortisol regulation (Adam et al., 2017). Others have employed psychosocial stress (using the Trier social stress test) for inducing cortisol elevation *via* social stress to examine its influences on IGT decision-making (e.g., van den Bos et al., 2009; Wemm and Wulfert, 2017). However, inducting social stress shows heterogeneous cortisol response attributed to the procedural variations in inducing social stress (Liu et al., 2017). Therefore, awakening cortisol provided a naturally occurring diurnal measure of cortisol response and regulation. Apart from diurnal regulation of cortisol, breath counting was used to enhance cortisol regulation (Ma et al., 2017) due to its working memory benefits (Levinson et al., 2014) and was compared with number counting that provided no cortisol-reducing or working memory benefits (Garavan, 1998). A consistent version of IGT at baseline and retest provided task repetition that benefits IGT decision-making in males (Bechara et al., 2000; Overman and Pierce, 2013). Cortisol and testosterone were expected to account for male IGT decision-making in a phase-specific manner, with cortisol regulation enhancing the hormones’ phase-specific effects.

## Materials and Methods

### Participants

Forty-two healthy right-handed male participants (mean age = 23.37 years, SD = 3.89) volunteered for the study. A power analysis (G power) suggested that using a sample size of 36 would be sufficient to reach the desired power (0.95) and large effect size (0.70). The participants were recruited as part of a study on mind, body, and cognition. The inclusion criteria were as follows: >18 years old, medication-free, no history of psychiatric or respiratory illness, and willing to comply with the protocol for saliva sample analysis (i.e., early morning empty stomach collection of the saliva sample before and after the counting procedure). Participants in the two groups of counting type were matched in terms of sex (all male), age (breath counting = 22.39 years, number counting = 23.82 years), handedness (right-handed), education (undergraduate engineering program), and comfort with the English language.

### Measures

#### Psychology Experiment Building Language

The psychology experiment building language was used to assess decision-making in the IGT. This task assesses decision-making where the participant has to choose between short-term, risky card decks (decks A and B) and long-term, safe reward decks (decks C and D). Unknown to the participant, the task consists of 100 trials (1 trial = 1 card drawn, 20 trials = 1 block). Long-term decision-making is reflected in a score that is calculated by taking the number of cards drawn from the safe decks minus those drawn from the risky decks [(C + D) − (A + B), calculated for 20 trials]. The net score of the first 40 trials reflects decision-making in the uncertainty phase because the choice outcomes are relatively unknown in the initial trials; the net score of the last 40 trials reflects decision-making in the risk phase because the choice outcomes are known in the later trials (Brand et al., 2007). A high net score reflects drawing fewer cards from the risky immediate reward decks and drawing more cards from the safe reward decks. Decision-making net scores were examined before and after counting (IGT 1 and IGT 2). Unknown to the participants, the IGT version was kept consistent for the two sessions. High variability in decision-making is observed despite increased task exposure (e.g., Buelow et al., 2013; Lin et al., 2013; Bull et al., 2015). Nearly 54% of the participants formed stable preferences within the 100 trials, whereas 28% did not develop a preference even after 200 trials (Bull et al., 2015). The complex nature of the IGT makes it suitable for maintaining a consistent IGT version in two testing sessions.

#### Cognitive and Impulsivity Measures

Because risk-taking in the IGT reflects cognitive and motor impulsivity (Bechara et al., 2000), two additional measures were included: (a) the digit span task (forward; Wechsler, 2008) that assesses the cognitive component, specifically working memory, by asking participants to recall digits (1–9), presented in increasing order (poor performance in the digit span task co-occurs with poor IGT decision-making; e.g., Zhou and Ni, 2017), and (b) the Simon task (Simon, 1990) that assesses motor impulsivity due to stimulus–response incongruence. A stimulus (colored circle) appears and the participant responds *via* button press (red = left side, blue = right side). It is observed that the response time is higher when the stimulus color is incongruent to the response side (i.e., red color stimulus appears on the right side). Simon task performance requires inhibiting the response that is based on the target location. Males show faster motor inhibition in the Simon task (Evans and Hampson, 2015), and motor inhibition seems to facilitate regulatory control in IGT decision-making (van den Wildenberg and Crone, 2005). The digit span task assessed the cognitive component and the Simon task assessed the motor component of impulsivity. The tasks were assessed before and after counting, maintaining consistency, and order of the tasks on the two testing occasions.

### Procedure

The ethics committee of the institution approved the protocol, and all participants provided signed informed consent. Participants received payment for participation at the end of the study (500 INR). All research was carried out in accord with the principles expressed in the Declaration of Helsinki. Because cortisol rises to 50% within 30 min of awakening and starts to decline thereafter, but remains elevated for more than 60 min (Wust et al., 2000), the participants were requested to arrive within 30 min of awakening on an empty stomach (the study venue was close to the male hostel to enable timely arrival, and the participants were provided breakfast at the end of the session). The study followed regulations for saliva testing; for example, all participants were tested within 45 min of awakening to obtain peak basal hormone levels and within the duration of early morning cortisol surge (7:00–10:00 a.m.), ensuring that the tests were carried out on an empty stomach. Participants were tested in groups of 6−10 and were assigned to two groups (counting type) using an odd–even scheme. An equal number of participants were tested in the two groups in a day, and the same laboratory space was used to ensure homogeneity in the acclimatization of both groups. After obtaining demographic information, the participants performed the digit span task, followed by the Simon task and the IGT (IGT 1). After this baseline assessment of the tasks, the participants provided their first saliva sample (saliva T1) and proceeded to the counting session. They were assigned to either one of the two counting groups using an odd–even scheme (e.g., odd number participant assigned to the breath counting group and an even number participant assigned to the number counting group; see counting instructions and study design flow in Supporting Information). All participants gave saliva samples and performed the IGT and other tasks before and after counting. Task performance was examined under peak concentrations of the hormones (cortisol and testosterone) and cortisol decline. This procedure is similar to that of Stauble et al. (2013), where task assessment (T1) was followed by a saliva sample pre- and post-stress alteration (i.e., counting in the present study), followed by an assessment of task performance (T2). The participants were seated on mats and performed mental counting as per their assigned condition (i.e., breathe or number counting). Immediately after the counting session, the participants provided the second saliva sample (saliva T2) and performed the working memory task and the Simon task with the IGT (IGT 2; see Supplementary Figure 1). The participants completed the protocol and were remunerated for their participation at the end of the study.

### Saliva Sample

Each participant provided saliva samples on an empty stomach, and the samples were collected between 7:00 and 10:00 a.m. Analyses of the first two saliva samples, before and after the counting procedure (breath *vs*. number counting), are reported. Each sample of up to 3 ml was collected in separate sterilized vials. The vials were labeled and stored in a cold storage box. All vials in the cold storage box were transported to a pathology laboratory within 3.5 h, where saliva analysis was performed according to the guidelines set forth by Schultheiss and Stanton (2009). Samples were stored at −20°C until the assay was performed. For the analysis, all samples were removed from the freezer, allowed to thaw completely at room temperature, and then thoroughly mixed. An aliquot of each sample was placed into a centrifuge for 10 min at 2,000 × *g* in an attempt to produce a clean supernatant, which was then used for examination. The samples were centrifuged according to the guidelines for electrochemiluminescence immunoassays. According to the manufacturer, the detection range for cortisol was 0.20–75 ng/dl, while the coefficient of variation was 6.5%. The range for testosterone was 10–1,500 ng/dl (0.35–52.1 nmol/L), while the coefficient of variation was 7.6%. The cortisol concentrations (in micrograms per deciliter) displayed a non-normal distribution, so a log10 transformation was performed. Cortisol levels at T1 and T2, average cortisol level (average of T1 and T2), and cortisol decline (T1 minus T2), as cortisol measures, were used for analysis. Testosterone was analyzed from the first saliva sample and was non-normally distributed and log-transformed (log10; Stanton et al., 2011). The data of two participants were excluded based on the outlier detection method of 3 standard deviation (mean ± 3 SD; Mehta et al., 2015): one participant was excluded based on testosterone (mean = 2.1018, SD = 0.55626, 3 SD = 1.66878, range = 0.43302–3.77058, and outlier = −0.10) and another participant excluded based on the difference in cortisol (cortisol T1 minus cortisol T2; mean = 0.0730, SD = 0.12404, 3 SD = 0.37212, range = −0.29912–0.44512, and outlier = 0.53).

### Statistical Analysis

All data were imported into statistical software for social sciences version 18. The threshold for significance was 0.05. A mixed model analysis of variance used within-subjects variables (e.g., pre- and post-counting cortisol and pre- and post-counting task performance) and between-subjects variables (e.g., counting type) to examine changes in cortisol and IGT decision-making and other measures (working memory and inhibition tasks). Participants gave saliva samples and performed the IGT task before and after counting. Counting type was the between-group variable (i.e., pre- and post-counting IGT performance was compared between the two groups, breath counting and number counting) and repeated saliva samples and pre–post IGT task scores were treated as repeated measures or within-subjects measures (i.e., these measures were assessed repeatedly, providing a within-subject comparison). In support of a recent call to report statistical results of hormone data that enable examining the effect of outlier exclusion (Pollet and van der Meij, 2017), the analyses are repeated with outlier exclusion. Four hierarchical regression analyses were used to understand how a change in IGT choices might be accounted for by hormones. The contributions of cortisol, testosterone, and their interaction to a change in IGT decision-making were examined. The cortisol and testosterone values were mean-centered, and two separate interaction terms were derived for cortisol and testosterone interaction: (a) average cortisol × testosterone and (b) cortisol decline × testosterone. The first analysis aimed to predict change in decision-making in the uncertainty phase: average cortisol was entered at step 1, testosterone was entered at step 2, and cortisol × testosterone interaction was entered at step 3. A similar analysis was used for decision-making in the risk phase. Next, two analyses were performed using cortisol regulation (cortisol decline) at step 1, testosterone at step 2, and cortisol × testosterone interaction at step 3 for the uncertainty and the risk phase. Bootstrapped coefficients with 95% confidence intervals (CIs) and bias-corrected values (2,000 samples) are reported (significance level reported for 0.05, and *p* values less than 0.10 were reported to indicate a trend).

## Results

Cortisol decline was analyzed using pre- and post-counting cortisol levels as a within-subjects variable and counting type (breath and number) as a between-subjects variable. The main effect of cortisol was significant [*F*(1, 40) = 15.27, *p* < 0.0001, partial *η*^2^ = 0.28], suggesting cortisol decline (mean 1 = −0.49, mean 2 = −0.56). The two-way interaction of cortisol and counting type was not significant, indicating that counting type had no effect on cortisol decline [*F*(1, 40) = 1.79, *p* = 0.19].

### Alteration in Decision-Making and Working Memory

Decision-making in the uncertainty trials (trials 1–40 for IGT 1 and IGT 2) was a within-subjects variable and counting type was the between-subjects variable. The results showed that the main effect of the IGT scores in the uncertainty phase was significant [*F*(1, 40) = 4.37, *p* = 0.043, partial *η*^2^ = 0.10], indicating increased long-term decision-making (mean 1 = −0.86, mean 2 = 3.13). The non-significant two-way interaction suggested that the change was unaffected by counting type [*F*(1, 40) = 0.003, *p* = 0.96]. The results for decision-making in the risk phase (net scores for trials 60–100 for IGT 1 and IGT 2) showed that the main effect was non-significant [*F*(1, 40) = 1.62, *p* = 0.211] and decision-making in the risk phase remained unchanged (mean 1 = 8.82, mean 2 = 6.18). Two-way interaction with counting type was not significant [*F*(1, 40) = 1.50, *p* = 0.23].

The malleability of decision-making in the uncertainty trials might be due to the greater demands on working memory in the uncertainty phase. Aligned with this expectation, the results from the working memory task performance (digit span task) showed a significant main effect of task performance [*F*(1, 40) = 9.42, *p* = 0.004, partial *η*^2^ = 0.19], suggesting improved working memory at retest (T2; mean 1 = 9.48, mean 2 = 10.97). Counting type had no effect [*F*(1, 40) = 0.24, *p* = 0.62]. Unlike working memory performance, the main effect of the Simon task scores was not significant [*F*(1, 40) = 0.10, *p* = 0.76], suggesting that inhibition did not change (mean 1 = 134.78, mean 2 = 134.98). The two-way interaction of counting type and Simon task scores was significant at the trend level [*F*(1, 40) = 3.05, *p* = 0.08, partial *η*^2^ = 0.07]. Breath counting marginally improved inhibition (mean 1 = 133.25, mean 2 = 134.55), and number counting lowered it (mean 1 = 136.32, mean 2 = 135.41). The results indicated post-awakening cortisol decline, and decision-making in the uncertainty phase showed improvement, potentially due to working memory.

### Effects of Cortisol–Testosterone on IGT Decision-Making

Four hierarchical regressions were used to examine the effects of cortisol, testosterone, and their interaction on decision-making (IGT net scores) in the uncertainty and the risk trials. Average cortisol (average of cortisol at T1 and T2), cortisol decline (cortisol at T1 minus T2), and testosterone were mean-centered. Average cortisol was uncorrelated with cortisol decline (*p* > 0.05), cortisol decline was uncorrelated with testosterone (*p* > 0.05), like others (Mehta and Josephs, 2010), and average cortisol and testosterone were marginally correlated (*r* = 0.29, *p* = 0.06). Two interaction terms were derived from cortisol and testosterone: (a) average cortisol × testosterone and (b) cortisol decline × testosterone. Counting type had no effect on the measures of interest and was excluded. The first two regressions used cortisol (average of cortisol) at step 1, testosterone at step 2, and cortisol and testosterone interaction was entered at step 3 to predict decision-making in the uncertainty phase and the risk phase examined separately (see the results in Table 1). Bootstrapped CIs with bias-corrected estimates are reported (2,000 samples).

**TABLE 1:** Results of hierarchical regression using average cortisol (step 1), testosterone (step 2), and average cortisol × testosterone (step 3) to explain Iowa gambling task (IGT) decision-making in the uncertainty and risk phases.

**Steps and predictors**		***R*^2^**	***F***	**Δ*R*^2^**	**Δ*F***	***B***	**SE**	**BCa 95% CI**
**DV = Uncertainty trials**								
1	CORT avg.	0.02	0.93 (1, 39)	0.02	0.91	–8.09	7.45	−23.33 to 13.27
2	CORT avg.	0.13	2.74 (2, 39)*	0.11	4.46**	−13.41**	6.85	**−27.46 to −2.80**
	Testosterone					9.30	6.46	-5.23 to 19.07
3	CORT avg.	0.14	1.88 (3, 39)	0.01	0.26	−12.83**	6.51	**−25.10 to −3.72**
	Testosterone					10.20	7.78	−4.53 to 21.95
	CORT avg. × testosterone					–10.70	33.92	−66.01 to 73.99
**DV = Risk trials**								
1	CORT avg.	0.01	0.46 (1, 39)	0.01	0.46	6.16	10.00	−9.46 to 37.40
2	CORT avg.	0.13	2.86 (2, 39)*	0.12	5.20**	0.00	8.52	−14.92 to 28.55
	Testosterone					10.76**	5.03	**1.30–18.60**
3	CORT avg.	0.13	1.86 (3, 39)	0.00	0.01	0.09	9.73	−16.61 to 37.81
	Testosterone					10.90**	5.43	**0.02–19.34**
	CORT avg. × testosterone					–1.66	24.93	−43.41 to 46.25

*Transformed and mean-centered predictors were entered in three steps: cortisol measures (CORT avg. = average of cortisol at T1 and T2), followed by testosterone, and lastly, average cortisol × testosterone interaction. *F* values are presented with degrees of freedom in parentheses. BCa bootstrapped values indicate 95% CI. Durbin–Watson values were within the recommended range of 1–3, suggesting independence of errors.*

Level of significance indicated as: *p < 0.10, **p < 0.05.

*Boldface values of BCa confidence intervals highlight a non-overlapping zero.*

The results for the uncertainty phase (regression 1) indicated that cortisol did not account for IGT decision-making (step 1) and testosterone (step 2) improved the model at trend-level significance [*F*(2, 39) = 2.74, *p* = 0.08], explaining 11% of the variance in decision-making in uncertainty (Δ*R*^2^ = 0.11, *p* = 0.04). The beta values indicated that cortisol impaired decision-making in the uncertainty trials [*B* = −13.41, *p* = 0.04, bias-corrected and accelerated (BCa) CI = −27.46 to −2.80], and testosterone’s effect was non-significant. Cortisol and testosterone interaction (step 3) did not improve the model fit (*p* = 0.15). The coefficient for cortisol indicated that it might be detrimental for decision-making in the uncertainty trials (*B* = −12.83, *p* = 0.04, BCa CI = −25.10 to −3.72). Testosterone’s effect and the effect of cortisol × testosterone interaction were non-significant.

The results for decision-making in the risk phase (regression 2) indicated that average cortisol did not account for decision-making (step 1) and testosterone (step 2) improved the model fit at trend-level significance [*F*(2, 39) = 2.86, *p* = 0.07], explaining 12% of the variance in decision-making (Δ*R*^2^ = 0.12, *p* = 0.03). The coefficients indicated that cortisol was non-significant, whereas testosterone improved decision-making in the risk phase (*B* = 10.76, *p* = 0.03, BCa CI = 1.30–18.60). The interaction term (step 3) did not improve the model fit (*p* = 0.15), and the coefficients indicated that cortisol’s effect was non-significant. Only testosterone improved decision-making in the risk trials (*B* = 10.90, *p* = 0.03, BCa CI = 0.02–19.34). The interaction of cortisol and testosterone was non-significant.

To examine the effect of cortisol regulation on male decision-making; cortisol decline was entered at step 1, testosterone at step 2, and the interaction of cortisol decline and testosterone was entered at step 3. Two separate regressions were carried out for decision-making in the uncertainty and risk phases (see results in Table 2). The results for the uncertainty phase indicated that cortisol decline (step 1) failed to account for decision-making. Adding testosterone (step 2) or the interaction of cortisol and testosterone (step 3) did not have a significant effect on the model fit (all *p* > 0.10). Beta values indicated that cortisol decline, testosterone, and their interaction did not influence decision-making in the uncertainty trials. Interestingly, cortisol’s negative effect on decision-making in uncertainty was not observed. Perhaps, cortisol’s impairment of decision-making in uncertainty might be eliminated with cortisol regulation.

**TABLE 2:** Results of hierarchical regression using cortisol regulation (step 1), testosterone (step 2), and cortisol decline × testosterone (step 3) to explain Iowa gambling task (IGT) decision-making in the uncertainty and risk phases.

**Steps and predictors**		***R*^2^**	***F***	**Δ*R*^2^**	**Δ*F***	***B***	**SE**	**BCa 95% CI**
**DV = Uncertainty trials**								
1	CORT dif.	0.01	0.54 (1, 39)	0.01	0.54	14.49	24.06	−41.47 to 55.21
2	CORT dif.	0.08	1.51(2, 39)	0.06	2.46	9.78	21.75	−35.17 to 40.33
	Testosterone					6.87	6.49	−5.57 to 15.83
3	CORT dif.	0.11	1.44 (3, 39)	0.03	1.28	3.25	30.68	−41.37 to 17.34
	Testosterone					5.31	7.13	−6.13 to 11.82
	CORT dif. × testosterone					42.19	178.51	−314.34 to 92.46
**DV = Risk trials**								
1	CORT dif.	0.05	0.46 (1, 39)	0.00	0.08	5.88	32.73	−53.67 to 55.54
2	CORT dif.	0.13	2.86 (2, 39)*	0.13	5.64**	–1.53	26.38	−49.38 to 31.84
	Testosterone					10.81**	5.10	**1.97–17.67**
3	CORT dif.	0.25	4.02 (3, 39)**	0.12	5.63**	15.02	23.56	−56.44 to 12.11
	Testosterone					7.59*	4.09	**0.61–12.61**
	CORT dif. × testosterone					87.18**	91.56	−143.57 to 185.72

*Transformed and mean-centered predictors were entered in three steps: cortisol regulation (CORT dif. = cortisol decline assessed as cortisol at T1 minus that at T2) followed by testosterone, and lastly the cortisol decline x testosterone interaction. *F* values presented with the degrees of freedom in parentheses. BCa bootstrapped values are 95% CIs. Durbin–Watson values were within the recommended range of 1–3, suggesting independence of errors.*

Level of significance indicated as: *p < 0.10 and ** p < 0.05.

**FIGURE 1:**
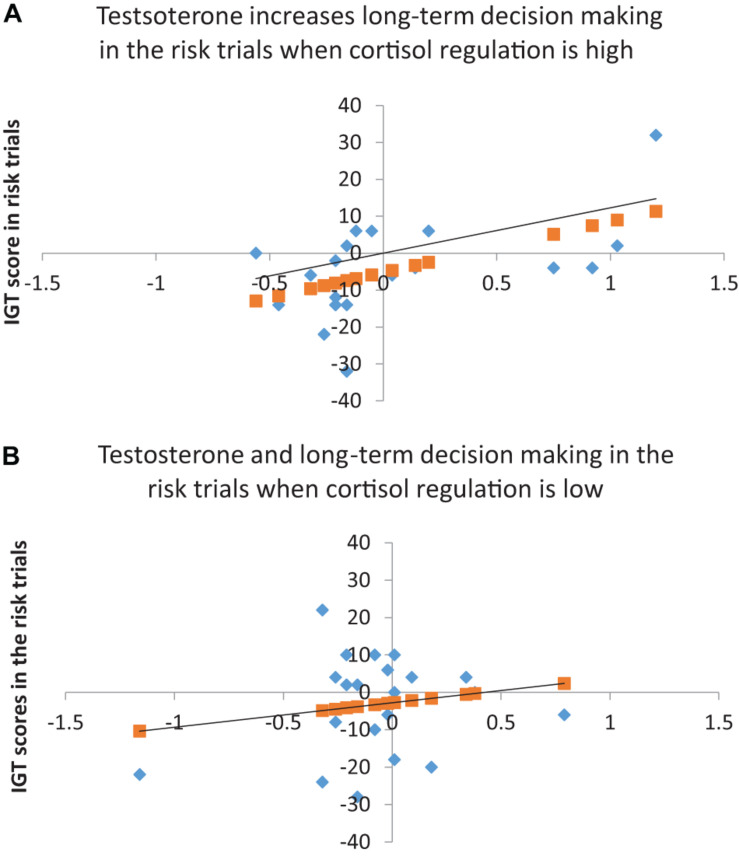
**(A)** Testosterone and long-term decision-making in the risk trials when cortisol regulation is high (*R*^2^ = 0.29, *p* < 0.05). **(B)** Testosterone and long-term decision-making in the risk trials for low cortisol regulation (*R*^2^ = 0.03, *p* > 0.05). Linear regression line shows the predicted long-term choices in the risk trial (*Y*).

The results for the risk phase indicated that cortisol decline (step 1) did not account for decision-making and testosterone (step 2) improved the model fit at trend-level significance [*F*(2, 39) = 2.86, *p* = 0.07], explaining 13% of the variance in decision-making (Δ*R*^2^ = 0.13, *p* = 0.02). The coefficients indicated that cortisol decline did not have an effect and testosterone significantly improved decision-making (*B* = 10.81, *p* = 0.04, BCa CI = 1.97–17.67). Adding cortisol decline and testosterone interaction (step 3) improved the model fit significantly [*F*(2, 39) = 4.02, *p* = 0.01), explaining 12% of the variance in decision-making (Δ*R*^2^ = 0.12, *p* = 0.02). The coefficients indicated that cortisol decline had no effect on decision-making (*p* > 0.10) and testosterone improved decision-making in the risk phase (*B* = 7.59, *p* = 0.06, BCa CI = 0.61–12.61). Although the coefficients for the interaction of cortisol decline and testosterone showed significant improvement in decision-making in risk (*B* = 87.18, *p* = 0.03, BCa CI = −143.57–185.72), the CIs overlapped with zero, suggesting that the interaction effect might not be robust. A joint regulation of cortisol and testosterone might have influenced male risk-taking; therefore, the interaction of cortisol decline and testosterone was further examined to determine whether testosterone’s (predictor) effect on decision-making in the risk phase (dependent variable) varied with high and low levels of cortisol regulation (median-based cutoffs). The analysis indicated that testosterone improved decision-making in high cortisol decline (*β* = 13.79, *p* = 0.017), but testosterone’s effect on decision-making in low cortisol decline was non-significant (*p* > 0.10). High cortisol regulation (decline) might contribute to testosterone regulation and benefit male long-term decision-making in the risk phase (see Figure 1).

Together, the results of four regressions indicate that cortisol and testosterone might account for male decision-making in the IGT. Cortisol might impair decision-making in the uncertainty trials (regression 1), whereas testosterone might improve decision-making in the risk trials (regression 2). Cortisol regulation (decline) might eliminate the detrimental effects of cortisol on decision-making in the uncertainty trials (regression 3), and high cortisol regulation might aid testosterone’s effects to improve long-term decision-making in the risk trials (regression 4).

## Discussion

The study aimed to understand the male low-risk, long-term decision-making in the IGT by examining the potential contributions of cortisol, testosterone, and their interaction to uncertainty and risk as two phases of the IGT. Decision-making was assessed at the circadian point of peak testosterone and high cortisol regulation (i.e., post-awakening cortisol elevation and decline) using breath counting as a cortisol regulation measure with cognitive benefits. The results confirmed cortisol decline in the all-male sample of the present study. Blunted cortisol decline in healthy participants is characteristic of early life stressors (Kuras et al., 2017) and is considered particularly maladaptive in males (Carol et al., 2016). Furthermore, the results indicated that cortisol decline was independent of counting type. Others have also observed that, contrary to expectations, a breathing-based intervention failed to influence cortisol reduction (Engert et al., 2017). Cortisol regulation in male IGT decision makers might not be malleable to short-term interventions such as breath counting. Similarly, IGT decision-making in uncertainty and risk was unaffected by counting type, and the results align with an earlier observation of male IGT decision-making being non-malleable to short-term interventions (Singh and Mutreja, 2020).

Decision-making was altered in a phase-specific manner, and the results indicated that decision-making was altered in the uncertainty phase, whereas it remained stable in the risk phase. Working memory task performance showed alterations, whereas inhibition task performance remained consistent. The cognitive demands of decision-making in the uncertainty phase are different from those in the risk phase, and the results from the working memory task align with this assumption. The change in the uncertainty phase might be linked to working memory (digit span task), indicating that decision-making in uncertainty relies on working memory (Bagneux et al., 2013). Furthermore, working memory depletion influenced IGT decision-making (Hinson et al., 2002), which impaired decision-making only in healthy male controls (compared to substance dependence and conduct; Fridberg et al., 2013). Male decision-making in the uncertainty trials might be sensitive to cortisol-induced alterations in working memory (Preston et al., 2007; van den Bos et al., 2009).

Unlike the malleability in decision-making that was observed in the uncertainty phase, the results for the risk phase showed consistency in long-term decision-making. Male long-term decision-making is most prominent in the risk phase (e.g., van den Bos et al., 2009, 2013; Stanton et al., 2011; Evans and Hampson, 2014), suggesting that males tend to choose consistent long-term rewards in the risk phase rather than in the uncertainty phase. Others have also alluded to male decision-making being most resistant to change in the risk phase of the IGT (Buelow and Barnhart, 2018). Male long-term decision-making in the risk phase and motor inhibition in the Simon task performance were unaltered, indicating a possible link between inhibitory control and decision-making in the risk phase. Together, the results indicate a possible phase-specific distinction in male IGT decision-making: decision-making varied in the uncertainty trials potentially due to the link between cortisol activity and the working memory demands of the uncertainty phase, whereas decision-making remained consistent in the risk trials potentially due to the inhibitory demands of the risk phase.

Next, hierarchical regressions examined the effects of cortisol (cortisol average and cortisol decline), testosterone, and their interaction on male IGT decision-making in the uncertainty and risk phases. The results for the uncertainty trials indicated that cortisol did not account for IGT decision-making (step 1) and testosterone improved the model’s explanatory power (step 2), accounting for 11% decision-making in the uncertainty phase, and only cortisol’s effect was significant. The coefficients suggested that cortisol impaired decision-making in the uncertainty trials. These results align with those of other reports of cortisol impairment in male decision-making (Preston et al., 2007; van den Bos et al., 2009). Testosterone might improve our understanding of cortisol impairment in male decision-making in the uncertainty phase. The results could be attributed to the greater demand of long decision-making for cognitive resources (Lin et al., 2007; Singh and Khan, 2009), which aligns with other observations of specifically the uncertainty phase having higher demands on cognitive resources (Bagneux et al., 2013). Cortisol-impaired decision-making in the uncertainty phase suggests that IGT’s uncertainty and risk phases pose distinct demands, and testosterone might potentially account for the cortisol-induced impairment of male decision-making in the uncertainty trials.

The results of IGT decision-making in the risk phase showed that cortisol failed to explain decision-making (step 1), that adding testosterone improved the model fit at trend-level significance (step 2), and that only testosterone’s effects on decision-making were significant. The beta coefficients suggested that testosterone improved male decision-making in the risk phase. The cortisol and testosterone interaction model did not improve the model fit (step 3). Testosterone’s effect was prominent and testosterone improved decision-making in the risk phase. Testosterone tends to hamper long-term decision-making in the IGT risk phase (Reavis and Overman, 2001; Evans and Hampson, 2014). One possibility for testosterone improving decision-making in the present study might align with the dual-hormone hypothesis; peak cortisol activity (post-awakening response) potentially curtailed testosterone’s effect on male risk-taking (Mehta and Josephs, 2010).

Cortisol regulation (i.e., cortisol decline) was expected to accentuate the link between testosterone and decision-making in the risk phase because it might have high regulatory demand on testosterone. The results for the uncertainty phase indicated that cortisol regulation (step 1), testosterone (step 2), and cortisol regulation and testosterone interaction (step 3) failed to account for decision-making in the uncertainty phase.

The results for the risk phase indicated that cortisol regulation was not significant (step 1), that testosterone significantly improved the model fit (step 2), and the coefficient values indicated that only testosterone’s effect was significant and improved decision-making in the risk phase. The interaction of cortisol regulation and testosterone improved the explanatory power of the model (step 3). The coefficients indicated that only testosterone’s effect was significant such that it improved decision-making in the risk phase. A non-overlapping zero in the CIs suggested that the effect was reliable. Coefficients for the interaction of cortisol regulation and testosterone were significant; however, overlapping zero in the CIs suggests that the effect might be less reliable. A simple slope analysis was used to examine the interaction effect of cortisol regulation and testosterone. Testosterone improvement in decision-making in the risk phase was significant for high cortisol regulation. The dual-hormone hypothesis suggests that cortisol inhibits testosterone’s effects on male risk-taking (Mehta and Josephs, 2010); therefore, high cortisol regulation might facilitate testosterone regulation, and the dual regulation might navigate male decision-making in the risk phase toward safe, low-risk rewards.

Overall, the results of the first two regressions indicated that testosterone improved the model’s explanatory fit for decision-making in the uncertainty and risk phases, specifically the cortisol-impaired uncertainty phase decision-making, whereas testosterone benefitted the risk phase decision-making. Cortisol regulation accentuated the phase-specific effects of cortisol and testosterone, indicating that testosterone improved decision-making, and this improvement was evident only in the risk phase, where prominent effects of testosterone were expected. Combining cortisol decline and testosterone improved the model’s fit. The results lend support to cortisol’s and testosterone’s joint effects on male risk-taking (Mehta and Josephs, 2010; Knight et al., 2019). The results align with observations where stress and sex hormones accounted for male IGT decision-making in the risk phase (Alacreu-Crespo et al., 2019). Cortisol regulation improves cognitive control (Evans et al., 2012); possibly, it might have inhibited testosterone’s effect and promoted long-term decision-making in the risk phase.

## Limitations and Conclusion

The study explored the roles of cortisol and testosterone in male IGT decision-making. The results indicated that hormones might contribute to decision-making in a phase-specific manner; however, the results are preliminary, given the following limitations: the study utilized awakening surge in cortisol and testosterone to examine the effects of hormones at their peak levels and with immediate diurnal decline. Future studies could explore multiple points of diurnal decline in hormone concentrations across the day to examine whether cortisol’s and testosterone’s effects on male IGT decision-making alter through the day. Counting type failed to influence cortisol decline in male IGT decision makers. Although the study aimed to understand male decision-making, the inclusion of female participants would further our understanding of cortisol’s and testosterone’s effects on risk taking in IGT decision-making. The study used a within-subjects comparison of cortisol decline (pre and post); a larger sample size might enable between-subjects comparison of cortisol regulation and testosterone levels (high and low levels), and a larger sample size might benefit the marginally significant effects (trend-level significance, *p* < 0.10). There were no performance-dependent incentives in the task, potentially altering risk-taking; however, studies have documented that real monetary incentives did not alter IGT decision-making (Bowman and Turnbull, 2003). Although the participants were unaware that the task is being repeated (T1 and T2), a consistent task version might have contributed to improved task performance due to practice effect. However, as outlined earlier, due to the complex nature of the IGT, the performance did not show a uniform practice effect, instead showing phase-specific variations in decision-making (i.e., decision-making improved in the uncertainty trials and remained stable in the risk trials). Task consistency was maintained in the other tasks used in the present study (digit span and Simon task). The digit span task performance showed improvement, whereas the Simon task performance showed stability. Although the effects of hormones on IGT decision-making are explored in the present study, decision-making might have influenced the hormone levels. Future studies should examine whether improved reward learning and learning to accrue long-term rewards reduce cortisol, or whether poor learning and increased risk-taking increase testosterone. Despite the limitations, and the exploratory nature of the study, cortisol regulation and testosterone interaction explained 25% of the decision-making variation in the risk phase. Studies using hierarchical regression for IGT performance explain a modest proportion of IGT decision-making because IGT is a complex task with considerable heterogeneity (large standard deviations; Bowman and Turnbull, 2003; Newman et al., 2008; Singh and Khan, 2009; Singh, 2013a,b). For example, measures of emotional and cognitive intelligence explained 12% of IGT choices (adjusted *R*^2^ = 0.12; Ramchandran et al., 2020), personality explained 10% of the IGT choices (*R*^2^ = 0.10 for males and 0.05 for females; Hooper et al., 2008), and heart rate explained 19% of male IGT decision-making in the risk phase (Wemm and Wulfert, 2017).

### Significance

Testosterone and cortisol hormones might contribute to male IGT decision-making in a phase-specific manner; that is, testosterone might contribute to the cortisol-induced deficit in decision-making in the uncertainty phase and cortisol regulation might aid testosterone inhibition and enable safe decision-making in the risk phase. Others have attributed males’ preference for safe rewards to factors such as greater hemispheric specialization (Bolla et al., 2004) and high cognitive control in males (van den Bos et al., 2013). In an earlier study, we speculated that prominent male advantage in the risk phase of the IGT decision-making might reflect population-level testosterone (Singh et al., 2020). Although the results are preliminary, testosterone might contribute to male long-term decision-making in the risk phase of the IGT. Cortisol regulation potentially contributes to inhibiting testosterone, promoting long-term decision-making in males. Whether lower stress levels in males (Matud, 2004; Weekes et al., 2008) enable the regulation of cortisol and testosterone remains unclear. Males are overrepresented as household decision makers, specifically in gender-inequitable, developing societies with economic stress, so understanding the effects of stress and sex hormones on male decision-making might have broader implications for attaining the goals of gender parity. In male-dominated professions where decision-making takes place under stress, uncertainty, and risk (e.g., decision-making in nighttime military combat and high-risk medical emergencies), the effects of sex and stress hormones on decision-making might have vital implications.

## Data Availability Statement

The raw data supporting the conclusions of this article will be made available by the authors, without undue reservation.

## Ethics Statement

The studies involving human participants were reviewed and approved by Institute Ethics Committee, Indian Institute of Technology Delhi, New Delhi, India. The patients/participants provided their written informed consent to participate in this study. Consent to the publication of the results was taken in verbal and written form, assuring that it follows the conditions laid down by the Institute Ethics Committee (IEC).

## Author Contributions

The author confirms being the sole contributor of this work and has approved it for publication.

## Conflict of Interest

The author declares that the research was conducted in the absence of any commercial or financial relationships that could be construed as a potential conflict of interest.

## Supplementary Material

Supplementary Figure 1 Study design.
