# Supplementary material for: Role of Cortisol and Testosterone in Risky Decision-Making: Deciphering Male Decision-Making in the Iowa Gambling Task
Source: Front Neurosci. 2021 Jun 15;15:631195. doi: 10.3389/fnins.2021.631195 (PMC8239136; doi:10.3389/fnins.2021.631195)

Supporting Material

Group 1: Breath-counting instructions

In this part of the experiment, we want you to focus on your breath with your eyes closed, specifically on the process of inhaling and exhaling air. You are required to breathe in at a specific pace/speed, kindly following three paces/speeds in the following manner:

First, you will breathe at a very slow pace (demonstration). One breath consists of one slow inhalation and exhalation of air (1 count of breath). Count 20 slow-paced breaths, focusing only on your breathing. Indicate by raising your hand when you have finished counting these 20 slow-paced breaths.

Next, breathe at a medium pace (demonstration), counting a total of 40 such medium-paced breaths, focusing only on the breathing process. Indicate by raising your hand when you have finished counting these 40 medium-paced breaths.

Finally, breathe at a fast pace (demonstration), counting 40 of such fast-paced breaths. Indicate by raining your hand when you have finished counting the last fast-paced breath.

For all three types of breath counting, please keep your mind/attention only on the breathing process (inhaling and exhaling of breath), maintaining your eyes closed throughout.

Please do this three-pace breath counting process (i.e., cycle 1: count 1 – 20 in slow pace, count 1 – 40 in medium pace, and count 1 – 40 in fast pace breathing) three times. Please ask any questions or express any concerns you may have.

Group 2: Number-counting instructions

In this part of the experiment, we want you to mentally count numbers, keeping your eyes closed, and specifically focusing your attention on the number-counting task. You are required to count the numbers silently in your mind at a specific pace/speed, following three paces/speeds in the following manner:

First, you will count from 1 – 20 in your mind at an extremely slow pace (demonstration), starting with the number ‘1’, followed by ‘2’, ‘3’, until you have counted to the number ‘20’ in your mind. Count the numbers at a slow pace, focusing your mind/attention solely on the numbers. Indicate by raising your hand when you have finished counting to the last number, 20, at a slow pace.

Next, count from 1 – 40 in your mind at a medium pace (demonstration), starting with the number ‘1’, followed by ‘2’, ‘3’, until you have counted the number ‘40’. Count the numbers at a medium pace, focusing your mind/attention only on the numbers. Indicate by raising your hand when you have finished counting to the last number, 40, at a medium pace.

Finally, you will count from 1 – 40 in your mind at a fast pace (demonstration), starting with the number ‘1’, followed by ‘2’, ‘3’, until you have counted to the number ‘40’. Count the numbers at a fast pace, focusing your mind/attention only on the numbers. Indicate by raising your hand when you have finished counting to the last number of 40 at a fast pace.

For all three types of number counting in your mind, please keep your mind/attention only on the number and maintain your eyes closed throughout.

Please perform this three-pace number counting task (i.e., cycle 1: count 1 – 20 in slow pace, count 1 – 40 in medium pace, and count 1 – 40 in fast pace counting) three times. Please ask any questions or express any concerns you may have.

Supplementary Figure. Study design

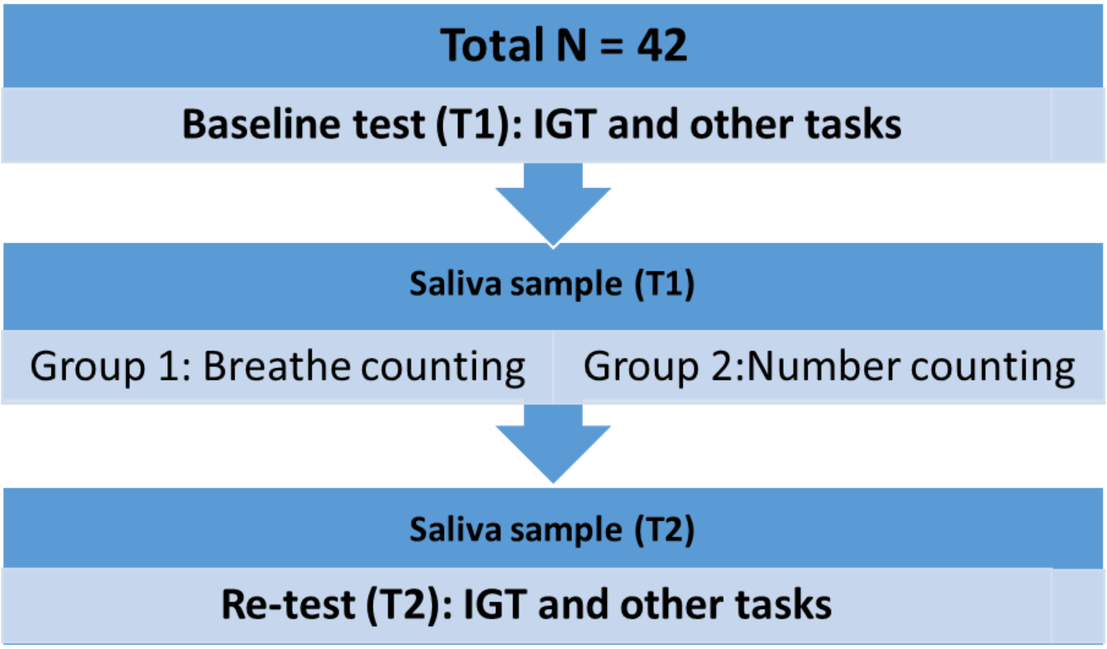

Supplement: Supplementary Figure 1 — Study design. [file Presentation_1.pdf]
